# Salmagundi

**Published:** 1885-07

**Authors:** 


### SALMAGUNDI.

EDITED BY E. G. BETTY, D. D. S.

THE export of "India" rubber from Brazil has increased very rapidly, amounting a couple of years ago to over 22,000,000 pounds. The expiration of the Cummins' patent *may* have had something to do with it.

IT is passing strange how a man, who owes you thirty cents, will have his attention suddenly attracted to the cornice of the tall block across the way, whenever you chance to meet him on the street.—*Pretzel's Weekly.*

"The June-bug has a wing of gold,
The Fire-fly, that of flame,
The Bed-bug hasn't any wings at all,
But he gets there all the same."

It is said that a company has been formed to enter into the "manufacture" of alumium upon a large scale; the method of producing the metal being so simple and inexpensive that it can be sold for $1.25 per pound.

Writers in the *London Lancet* call attention to the great value of hot water applications to the head in cases of fainting or syncope. They say also that a prompt use of it, applied to the forehead with cloths, will very often avert such attacks.

Dr. Jno. A. McClellan, of Gallipolis, Ohio, died at Bellaire, Ohio, while on his way to New York to engage in business with a dentist of that city. He was but 24 years of age at the time of his death, June 4th, and had just graduated at the Philadelphia Dental College, giving promise of being a fine operator and conscientious practitioner.

It is gratifying to see that each college, as it issues its annual announcement, devotes more or less space in announcing the action of the college Faculties Association at New York and Saratoga last summer. This Association is destined to work more good for the profession, begining in the near future, than any other single influence, for the reason that it is the result of concerted action, and the time has arrived when the sentiment of the propession, generally, demands *graduation* as the standard qualification for practice.

Our friend, Dr. N. S. Hoff, has lately taken upon himself the onerous and responsible duties of editorship, the name of the paper being the *Congregational Index*, and published in the interest of Congregationalism. He has just purchased a new pair of shears and contracted for a large supply of mucilage (now that the war in the Soudan is over), and when he comes across an item he thinks would adorn the columns of the *Index* he "pastes it in his hat" 'till its' time to publish it. "Angels and ministers of grace, defend us."

---

THE real change which produces old age is nothing more nor less than a slow but steady accumulation of calcareous matter in the system, which blocks the various organs and finally prevades the vital parts. People who desire to live long should avoid much bread, eat moderately, and satisfy the appetite with fruit, fish, poultry, young mutton, and beef, because they are least nitrogenous. Cistern water should be used instead of well or spring water, which contain more earthy salts. The solubility of these earthy salts is promoted by adding fifteen or twenty drops of diluted phosphoric acid to a glass of water.—*Surgical Reporter.*

---

*The Present* is the name of a new monthly magazine of "entertaining literature and practical information," published in Cincinnati, by J. M. Cochran & Co., and is successor to a weekly paper called the *Beehive.* It is in quarto form, printed on heavy laid paper with double column letter-press, most agreeable to the eye. Among the contributors to the June number (Vol. II., No. I.) may be mentioned Henry Hooper Esq., Assistant U. S. Prosecuting Att'y., Rev. B. W. Chidlaw the well known pioneer divine, Eliza Archard, formerly *E. A.*, of the Cincinnati Commercial, now of New York; Samuel F. Covington, Esq., identified with insurance interests, and others well known in the Ohio Valley. A prominent feature of the monthly is devoted to Building Associations and kindred matters.

J. M. Cochran & Co., 164 Race street, Cincinnati, Ohio, $1 per annum.

